# Simple Estimates for Local Prevalence of Latent Tuberculosis Infection, United States, 2011–2015

**DOI:** 10.3201/eid2410.180716

**Published:** 2018-10

**Authors:** Maryam B. Haddad, Kala M. Raz, Timothy L. Lash, Andrew N. Hill, J. Steve Kammerer, Carla A. Winston, Kenneth G. Castro, Neel R. Gandhi, Thomas R. Navin

**Affiliations:** Centers for Disease Control and Prevention, Atlanta, Georgia, USA (M.B. Haddad, K.M. Raz, A.N. Hill, J.S. Kammerer, C.A. Winston, T.R. Navin);; Emory University, Atlanta (M.B. Haddad, T.L. Lash, A.N. Hill, C.A. Winston, K.G. Castro, N.R. Gandhi)

**Keywords:** tuberculosis, latent tuberculosis, tuberculosis and other mycobacteria, Mycobacterium tuberculosis, bacteria, respiratory infections, infection, estimates, prevalence, public health surveillance, molecular epidemiology, United States

## Abstract

We used tuberculosis genotyping results to derive estimates of prevalence of latent tuberculosis infection in the United States. We estimated <1% prevalence in 1,981 US counties, 1%–<3% in 785 counties, and >3% in 377 counties. This method for estimating prevalence could be applied in any jurisdiction with an established tuberculosis surveillance system.

Approximately 25% of the world’s population is latently infected with *Mycobacterium tuberculosis*. Latent tuberculosis infection (LTBI) is an asymptomatic equilibrium between the immune response of the host and the infectious process. Although not infectious, LTBI can be activated years later as infectious tuberculosis (TB), which is why diagnosing and treating LTBI in high-risk populations is a key component of the World Health Organization End TB Strategy (*1**–**4*).

Most countries have established systems for surveillance of active TB. Public health interventions to control TB include timely detection and treatment of active cases and prompt investigations of persons with recent contact with someone who has infectious TB. However, few jurisdictions have estimates of local LTBI prevalence. Having such estimates could help direct TB prevention efforts for persons with the highest risk for infection, highest risk for progression to TB, and greatest benefit from treatment to prevent progression (*2‒4*). We describe a simple method that uses genotyping results from active TB cases to derive a population estimate of untreated LTBI prevalence for any jurisdiction.

## The Study

The US National TB Surveillance System contains 48,955 verified TB cases for 2011–2015. In the subset of 37,723 (77.1%) cases that were confirmed by culture, 36,104 (95.7%) had an *M. tuberculosis* isolate genotyped by the National TB Genotyping Service by using pacer oligonucleotide typing and 24-locus mycobacterial interspersed repetitive unit–variable number tandem repeat methods. The 50 US states and the District of Columbia are divided into 3,143 local jurisdictions (typically called counties). We used the US Census 2010 population denominator, annual TB incidence averaged during 2008–2015, and 2 assumptions for each county to derive an estimated prevalence of LTBI among residents. 

For the 1,360 counties with no genotyped TB cases, which corresponded to 8% of the US population, we estimated local LTBI prevalence as <1%. For other counties, we assumed that all genotyped TB cases not attributed to recent *M. tuberculosis* transmission arose from preexisting LTBI (i.e., were reactivation TB). We used the previously field-validated plausible source-case method (*5**–**7*) to attribute cases to recent transmission (i.e., plausible source case within 10 miles within previous 2 years having infectious TB and a matching genotype result) for the District of Columbia and 49 US states. All cases diagnosed in non–US-born persons within 100 days of entry into the United States were excluded because the presumption was that these persons did not represent infection acquired in the United States. Because some cases in Oklahoma were missing geographic identifiers for identifying the 10-mile radius, a modification for these cases in this analysis was that the plausible source case could have occurred anywhere in the same county. Our second assumption was that the same recent transmission versus reactivation TB proportions for genotyped cases would apply to nongenotyped TB cases in each county (*8*).

Based on the estimate of Shea et al. (*8*) of ≈0.084 cases of reactivation TB/100 person-years among US residents with LTBI, we applied a uniform population-level 0.10% annual risk for progression to active disease to derive an estimated number of county residents with LTBI. As sensitivity analyses, we examined how LTBI prevalence estimates would decrease with a higher 0.14% uniform annual risk and how estimates would increase with a lower 0.06% uniform annual risk. We present estimates as uncertainty limits and provide the formula and examples of this method (Table 1).

**Table 1:** Formula and examples of method for estimating prevalence of latent TB infection, United States, 2011–2015*

Variable	a	b	c	d	e	f	g	h
Jurisdiction	Population	Average annual no. active TB cases	Proportion of TB cases attributed to recent transmission	Annual no. cases attributed to reactivation TB	Estimated no. infected residents if 0.10% annual risk for progression	Estimated prevalence of infection if 0.10% annual risk for progression, %	Sensitivity analysis for estimated prevalence of latent infection, %	
Lower uncertainty limit based on 0.14% annual risk for progression	Upper uncertainty limit based on 0.06% annual risk for progression
Example X	Any size	0	NA	0	NA	<1	NA	NA
Example Y	150,000	1	0	1	1,000	0.7	0.5	1.1
Example Z	2,000,000	50	0.2	40	40,000	2.0	1.4	3.3

*Let a = jurisdiction population, b = average annual no. TB cases in that jurisdiction, and c = proportion of TB cases attributed to recent transmission (i.e., [1 – c] = proportion attributed to latent TB infection). Then if b = 0, d = 0, and f <1%, otherwise d = b × (1 – c) and e = d/0.0010 if one assumes a 0.10% annual risk and f = e/a (×100 to express as a percentage) or (d/0.0014/a for lower uncertainty limit and h = d/0.0006/a for upper uncertainty limit. NA, not applicable; TB, tuberculosis.

We estimated that 3.1% (uncertainty limits 2.2%–5.2% based on higher or lower risk progression assumptions) of the US population, corresponding to 8.9 (6.3‒14.8) million persons, were latently infected with *M. tuberculosis* during 2011–2015. County-level estimates varied widely: estimated LTBI prevalence of <1% in 1,981 counties, 1% –<3% in 785 counties, and >3% in 377 counties (Figure). As defined by the US Census Bureau Small Area Income and Poverty Estimates, poverty in >20% of the population was a characteristic of 146 (72%) of the 202 rural counties and 62 (35%) of the 175 metropolitan counties that had an estimated LTBI prevalence >3% (Table 2).

**Figure:**
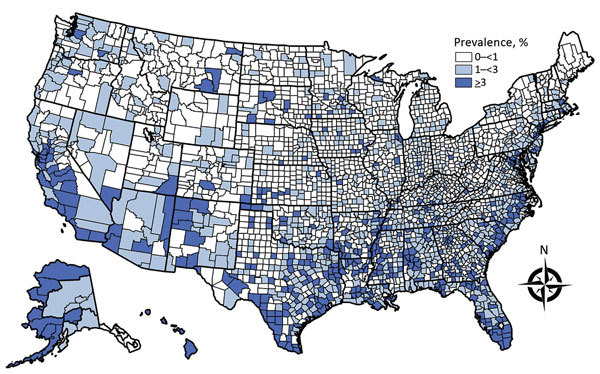
Estimated prevalence of latent tuberculosis infection, by county, United States, as derived from genotyped cases of tuberculosis reported to the US National Tuberculosis Surveillance System, 2011–2015. County equivalents (i.e., Alaska boroughs, District of Columbia, Louisiana parishes, and Virginia independent cities) are also shown. A modified method for analyzing data for Oklahoma is found in the text. Prevalence estimates for Alaska are aggregated by region.

**Table 2:** Characteristics of 1,976 rural and 1,167 metropolitan counties, by estimated prevalence of latent TB infection, United States, 2011–2015*

Characteristic	1,976 rural counties				1,167 metropolitan counties		
	1,454 with estimated prevalence <1%	320 with estimated prevalence 1%–<3%	202 with estimated prevalence >3%		527 with estimated prevalence <1%	465 with estimated prevalence 1%–<3%	175 with estimated prevalence >3%
US Census 2010 data							
Combined population of counties	28,727,127	11,750,121	5,816,158		37,414,210	115,341,399	109,697,523
Median county population, rounded to thousands	13,000	32,000	23,000		38,000	144,000	291,000
Estimated prevalence of *Mycobacterium tuberculosis* infection							
Estimated no. infected in all counties	126,140	191,707	329,547		212,563	2,300,435	5,772,136
Estimated median no. infected/county	0	500	1,112		124	2,376	12,388
County population living in poverty, %†							
<10	95 (7)	13 (4)	2 (1)		112 (21)	63 (14)	25 (14)
10–15.5	564 (39)	78 (24)	29 (14)		221 (42)	171 (37)	30 (17)
15.6–19.9	378 (26)	95 (30)	25 (12)		124 (24)	144 (31)	58 (33)
>20	417 (29)	134 (42)	146 (72)		70 (13)	87 (19)	62 (35)
Race/ethnic group in county with largest no. active TB cases reported							
Black non-Hispanic	81 (15)	42 (13)	60 (30)		45 (14)	86 (18)	57 (33)
White non-Hispanic	241 (45)	109 (34)	34 (17)		142 (44)	110 (24)	17 (10)
Hispanic	74 (14)	58 (18)	60 (30)		25 (8)	82 (18)	43 (25)
Alaska Native/Native American or Pacific Islander	36 (7)	14 (4)	15 (7)		8 (2)	8 (2)	3 (2)
Asian	43 (8)	24 (8)	8 (4)		48 (14)	118 (25)	46 (26)
No predominant race/ethnic group	979 (67)	73 (23)	24 (12)		259 (49)	61 (13)	9 (5)

*Values are no. (%) unless otherwise noted. County equivalents (i.e., Alaska boroughs, District of Columbia, Louisiana parishes, and Virginia independent cities) are also shown. US Department of Agriculture 2013 Rural–Urban Continuum Codes were dichotomized (i.e., codes 4–9 were considered rural and codes 0–3 were considered metropolitan). †County all-ages poverty level in 2011 as determined by US Census Bureau Small Area Income and Poverty Estimates.

## Conclusions

Preventing TB is a growing focus of TB control strategies in the United States and internationally. As governments, public health departments, and private sector partners intensify TB prevention activities, having a tool to understand local variations in LTBI prevalence could help prioritize resources (*2*–*4*).

We used routinely collected TB surveillance and genotyping data to derive untreated LTBI prevalence estimates for all US counties. This method was designed to be simple (Table 1). By excluding the contribution of any TB cases attributed to recent transmission, our estimates disregard the comparatively smaller number of recent infections and instead draw attention to more longstanding LTBI prevalence. Because time since initial *M. tuberculosis* infection was unknown, a uniform population-level 0.10% annual risk for progression to active disease was assumed. Changing that uniform risk to 0.14% would have decreased the number of counties with an estimated LTBI prevalence >3% to 113 counties. A change to 0.06% would have increased the number of counties with an estimated LTBI prevalence >3% to 516 counties.

A more sophisticated approach to estimate local longstanding LTBI prevalence might consider individual characteristics and differentiate risk for progression based on HIV status, age group, and possibly geographic region, place of birth, and recent migration (*8*). For example, a person receiving a TB diagnosis soon after arrival in a county would increase the LTBI prevalence estimates for that county, even if the TB was caused by an infection that had been acquired in another jurisdiction. Conversely, our overall estimate that 2.2%–5.2% of the US population is infected is similar to estimates from the 2011–2012 National Health and Nutrition Examination Survey (*9*).

For the United States, the last published nationwide county-level estimates of LTBI prevalence are based on 1958‒1965 data, when 275,558 men 17‒21 years of age who had lived their entire lives in 1 county were examined as they entered the US Navy (*10*). Men from poor counties in the southwestern United States and the Appalachian Mountains were more likely to have positive tuberculin skin test results (*10*). Compared with estimates from 5 decades ago, our estimates show a more diffuse pattern of higher LTBI prevalence counties (Figure). However, poverty remains a frequent characteristic of counties that we estimated as having a higher LTBI prevalence.

This method has limitations. We applied the proportion of genotyped TB cases in the county estimated to arise from preexisting LTBI to all nongenotyped TB cases in that county, which could overestimate the prevalence of LTBI in counties with many pediatric TB cases, which tend to be more difficult to confirm by culture techniques (i.e., cannot be genotyped), yet are sentinel events for recent transmission. Conversely, the genotyping methods used during 2011‒2015 might have overestimated recent TB infections (i.e., underestimated LTBI prevalence) in certain localities with longstanding genotyping clusters; this limitation should decrease as the National TB Genotyping Service transitions to universal whole-genome sequencing in 2018.

This method also has several advantages. It could be applied in jurisdictions without TB genotyping services, given an assumption or range of assumptions about the proportion of active TB cases arising from LTBI in the jurisdiction. Rather than relying on costly and imperfect LTBI screening methods, its starting point is verified cases of TB that are already routinely reported to established TB surveillance systems. If deemed applicable, an adjustment for underreported TB cases could be made. In addition, these cases represent infected persons who have the greatest risk for progression to active TB and are the populations most likely to benefit from interventions to prevent TB in the future.
